# ePrescribing-Based Antimicrobial Stewardship Practices in an English National Health Service Hospital: Qualitative Interview Study Among Medical Prescribers and Pharmacists

**DOI:** 10.2196/37863

**Published:** 2023-06-06

**Authors:** Kathrin Cresswell, Susan Hinder, Aziz Sheikh, Sarah Pontefract, Neil W Watson, David Price, Andrew Heed, Jamie Coleman, Holly Ennis, Jillian Beggs, Antony Chuter, Robin Williams

**Affiliations:** 1 Usher Institute The University of Edinburgh Edinburgh United Kingdom; 2 Institute of Clinical Sciences University of Birmingham Birmingham United Kingdom; 3 Newcastle upon Tyne Hospitals NHS Foundation Trust Newcastle United Kingdom; 4 Institute for the Study of Science, Technology and Innovation The University of Edinburgh Edinburgh United Kingdom

**Keywords:** antimicrobial resistance, antimicrobial stewardship, electronic prescribing, hospitals

## Abstract

**Background:**

Antimicrobial resistance, the ability of microorganisms to survive antimicrobial drugs, is a public health emergency. Although electronic prescribing (ePrescribing)–based interventions designed to reduce unnecessary antimicrobial usage exist, these often do not integrate effectively with existing workflows. As a result, ePrescribing-based interventions may have limited impact in addressing antimicrobial resistance.

**Objective:**

We sought to understand the existing ePrescribing-based antimicrobial stewardship (AMS) practices in an English hospital preceding the implementation of functionality designed to improve AMS.

**Methods:**

We conducted 18 semistructured interviews with medical prescribers and pharmacists with varying levels of seniority exploring current AMS practices and investigating potential areas for improvement. Participants were recruited with the help of local gatekeepers. Topic guides sought to explore both formal and informal practices surrounding AMS, and challenges and opportunities for ePrescribing-based intervention. We coded audio-recorded and transcribed data with the help of the Technology, People, Organizations, and Macroenvironmental factors framework, allowing emerging themes to be added inductively. We used NVivo 12 (QSR International) to facilitate coding.

**Results:**

Antimicrobial prescribing and review processes were characterized by competing priorities and uncertainty of prescribers and reviewers around prescribing decisions. For example, medical prescribers often had to face trade-offs between individual patient benefit and more diffuse population health benefits, and the rationale for prescribing decisions was not always clear. Prescribing involved a complex set of activities carried out by various health care practitioners who each only had a partial and temporary view of the whole process, and whose relationships were characterized by deeply engrained hierarchies that shaped interactions and varied across specialties. For example, newly qualified doctors and pharmacists were hesitant to change a consultant’s prescribing decision when reviewing prescriptions. Multidisciplinary communication, collaboration, and coordination promoted good AMS practices by reducing uncertainty.

**Conclusions:**

Design of ePrescribing-based interventions to improve AMS needs to take into account the multitude of actors and organizational complexities involved in the prescribing and review processes. Interventions that help reduce prescriber or reviewer uncertainty and improve multidisciplinary collaboration surrounding initial antimicrobial prescribing and subsequent prescription review are most likely to be effective. Without such attention, interventions are unlikely to fulfill their goal of improving patient outcomes and combatting antimicrobial resistance.

## Introduction

Antimicrobial resistance, defined as the ability of microorganisms to survive antimicrobial drugs, is a serious and increasing public health threat with an estimated 1.3 million deaths in 2019 worldwide [1]. Improving antimicrobial prescribing behavior has the potential to contribute to efforts to reduce antimicrobial overuse [2,3]. The issue has been exacerbated by the COVID-19 pandemic, where, during initial waves, an estimated 70% of hospitalized patients were prescribed antimicrobials as precaution against potential bacterial coinfections [4].

Strategies to mitigate antimicrobial resistance are now central to UK and international population health policies, promoting the “Five Rights” of medication use: the right patient, the right drug, the right time, the right dose, and the right route [5]. However, ways to achieve this gold standard are far from clear. One promising approach to improve prescribing behavior and reduce antimicrobial consumption is the development of multifaceted antimicrobial stewardship (AMS) programs involving a combination of technological and behavioral interventional components [6]. These are designed to educate all health care practitioners around good AMS practices, to educate medical prescribers in appropriate antimicrobial use (eg, reducing the inappropriate use of broad-spectrum antimicrobials, optimization of doses or durations, regular review of antimicrobial prescriptions, and deprescribing or stopping antimicrobials), and to encourage adherence to guidelines. These AMS interventions are frequently supported by specific interactions within computerized physician order entry (CPOE) and clinical decision support (CDS) systems (Textbox 1) [7-9].

**Computerized physician order entry (CPOE) and clinical decision support (CDS) systems to promote antimicrobial stewardship.**

- CPOE: to improve the accuracy of prescribing, the CPOE offers clinicians sets of antimicrobials that are appropriate for the indication. The sets include the defined daily dose and days of therapy. These can be aligned to local antimicrobial stewardship guidelines or consultant preferences.
- CDS systems provide clinicians with knowledge stored electronically to assist in prescribing antimicrobials appropriately.

However, despite much research and high hopes for the implementation of AMS programs, many have failed to show significant impacts on antimicrobial prescribing [10]. A key obstacle is the way new technological interventions fail to integrate with existing practices and workflows, resulting in prescribers ignoring procedures and overriding alerts [11,12]. For example, a controlled pre-post study that introduced a CDS-based requirement to input the indication for an antimicrobial prescription did not result in more appropriate prescribing [13]. Subsequent qualitative interviews with prescribers showed that indications entered into the system were not followed up because the antimicrobial approval processes interrupted workflows. This is supported by qualitative studies highlighting incompatibility of ePrescribing-based software with existing workflows of health care staff as a key barrier to adoption [14,15]. Conversely, AMS-based interventions that align with the existing workflows tend to show stronger impact on AMS practices [16].

There is a need to explore the existing prescribing practices and workflows to design systems that are more effectively used to improve AMS and thereby reduce the risk of antimicrobial resistance. In this study, we aim to understand what technology-based AMS practices are used by prescribers in their clinical practice, including existing workflows, and explore how these could be better supported. We plan to use this information to develop and optimize a new ePrescribing-based intervention to improve AMS in the same hospital.

## Methods

### Study Design and Setting

We conducted a qualitative semistructured interview study in an acute National Health Service (NHS) hospital (Textbox 2) in England using one of the main ePrescribing systems in use, to obtain opinions and insights regarding AMS and prescribing practices.

**Acute hospital context.**

Hospital A is a large National Health Service (NHS) Foundation hospital in England. It has 1800 beds and 17,000 staff across several hospital sites. It is considered “digitally mature” having reached Global Digital Exemplar status within NHS England.

The hospital has an electronic prescribing (ePrescribing) system implemented as part of an enterprise system. The system at the study site does not have any “built-in” antimicrobial stewardship (AMS) functionality switched on for users. At the time of this study, the hospital ePrescribing system had the following functionality:

- Procedure-specific order sets in vascular surgery
- Access to locally developed antimicrobial prescribing guidelines
- A nationally developed and integrated Antibiotic Review Kit [17], which included educational material for prescribers and required them to indicate a possible (suspected) and probable (confirmed by microbiology) infection
- The option for the prescriber to include a review date for antimicrobials
- A free text indication field
- Stop dates indicating when an antimicrobial is to be stopped (no stop alerts).

The AMS policy of the hospital includes education of newly qualified doctors and access to pharmacy and microbiology departments for advice. Electronic medical and nursing documentation was added to the ePrescribing system a few months before data collection.

### Sampling and Recruitment

We purposefully sampled pharmacists and doctors with a range of seniority and experience, who worked on wards that have a high use of antimicrobials in the hospital and that were to adopt the developed intervention [18]. In doing so, we included participants who were involved in the prescribing and reviewing of medicines. We excluded those who were not involved in these processes. Participants were recruited with the help of gatekeepers, including the lead informatics pharmacist, the chief pharmacist, the lead consultant in infectious diseases, and the AMS pharmacist. They were asked if they were interested in participating in an interview by gatekeepers. If participants expressed interest, their contact details were passed on to the lead researcher (SH), who emailed them directly with the study information sheet and arranged an interview. We also snowball sampled further participants by asking interviewees to recommend others [19], actively searching for a range of different opinions on AMS practices and electronic prescribing. We stopped recruitment when no significant new themes were emerging during the concurrent analysis (saturation). Overall, we invited 34 participants via email. Sixteen of these did not respond to our invitation (which included a reminder email approximately a week later).

### Data Collection

One-to-one interviews were conducted remotely over Microsoft Teams by SH using a semistructured interview guide between February 9, 2021, and February 14, 2022 (Textbox 3). The questions explored existing prescribing and AMS practices, workflows and team relationships, as well as potential areas for improvement. The researcher (SH) used prompts and tailored the questions to the role of the interviewee and also to emerging topics. Interviews were audio-recorded and transcribed verbatim by a professional transcriber. Each interview lasted between 30 and 60 minutes.

**Indicative interview guide.**

- What is your role?
- What is your role in antimicrobial prescribing?
- What are the typical processes and work practices related to antimicrobial prescribing? Who else is involved and how?
- What guidelines are in place?
- Are there differences between how things are meant to be done formally and how things actually happen?
- What are the practices of antimicrobial stewardship (AMS) in your ward or hospital? Does it differ from ward to ward?
- What barriers to AMS do you perceive there to be in your ward or hospital? In general?
- What are your experiences stopping, switching antimicrobials and changing from intravenous to oral?
- If you could, is there any aspect of the review process that you would want to change?
- What are your experiences with moving patients to outpatient parenteral antimicrobial therapy in your ward or hospital?
- What are the strengths and weaknesses of how things are done at the moment? If you could, is there anything you would change?
- What are your experiences with using the system for prescribing and reviewing antimicrobials? Do you have experience with other ePrescribing systems?
- If you were to describe an “ideal” intervention for AMS that would combine the ePrescribing system and work practices, what would it look like?
- How have things changed since the start of the COVID-19 pandemic in terms of antimicrobial prescribing, stewardship, and use of ePrescribing system?
- What do you think of any changes? What would you like to stay and what would you like to go?

### Data Analysis

The interview transcripts were coded using NVivo 12 (QSR International), applying a coding framework (Multimedia Appendix 1) developed by the research team. This was based on the existing literature and included themes surrounding barriers and facilitators to AMS. It also drew on the team’s background in sociotechnical analysis of ePrescribing, other digital health technologies, and the related Technology, People, Organizations, and Macroenvironmental factors framework, which helps to explore various ways in which technology and socio-organizational dimensions are interrelated [20]. Two additional members of the research team coded 2 different transcripts each to establish agreed themes and subsequent codes. We also allowed new themes to emerge during the analysis and explored tensions between respondents and contexts in most detail. The results were then fed back to the wider team, including gatekeepers, which resulted in minor modifications to the narrative but not in substantial changes to themes.

### Ethics Approval

We received ethical approval from the North of Scotland Research Ethics Service on November 18, 2019 (IRAS project ID 259104), and also received organizational approval from the participating hospital. Participants provided informed, audio-recorded, consent to participate. The setting and individual participants were anonymized to protect the anonymity of the participants.

## Results

We conducted 18 interviews (Table 1).

Our analysis identified three themes including (1) uncertainty of prescribers and reviewers around prescribing decisions, (2) prescribing as a set of activities enacted by multiple actors, and (3) hierarchies and relationships shaping prescribing and review practices. These will be explored in more detail in the paragraphs below.

**Table 1:** Characteristics of interviewees.

Profession	Gender	Age group (years)	Specialty
Doctor (clinical research fellow)	Female	30-40	Infectious diseases
Doctor (trainee)	Female	30-40	Renal and acute medicine
Consultant	Male	40-50	Infectious diseases and internal medicine
Consultant	Male	50-60	Pediatric hematology
Consultant	Female	50-60	Anesthetics and intensive care unit
Consultant	Male	50-60	Rheumatology
Consultant	Male	50-60	Neonatal medicine
Consultant	Female	50-60	Vascular surgery
Pharmacist	Male	50-60	Advanced clinical pharmacist
Pharmacist	Female	30-40	Clinical pharmacist
Registrar	Male	30-40	Infectious diseases and general medicine
Registrar	Male	30-40	Microbiology
Registrar	Male	30-40	Cardiology
Registrar	Male	30-40	Internal medicine
Various specialties	Male	20-30	Foundation year 1
Various specialties	Male	20-30	Foundation year 1
Various specialties	Female	20-30	Foundation year 2
Various specialties	Female	20-30	Foundation year 2

### Uncertainty of Prescribers and Reviewers Surrounding Prescribing Decisions

The prescribing and subsequent review of antimicrobials was characterized by uncertainty. For example, doctors described the clinical uncertainty over what to prescribe in individual cases, which often resulted in prescribing antimicrobials “just in case” because of fear of patients developing a serious infection. Respondents also reported that there was a tendency among medical prescribers to give priority to the patients in front of them rather than consider population health-related issues around antimicrobial prescribing, an issue that has been described as “competing necessities” [21].

“Most of the time then, we do just end up giving them something broad-spectrum, like Tazocin or something like that. We tend…I mean this is only personal experience, but people tend to err on the side of caution, as it were, not from a stewardship perspective but from a patient perspective.”

Participant 18, junior doctor

“I came from a general surgical job, and that was something that I definitely saw, was that it would depend which patient the consultant was under as to what antibiotics there was…in patients, who as far as we could see, were clinically the same, the same operations, the same risk factors, would have slightly different antibiotic prescriptions.”

Participant 5, junior doctor

Newly qualified doctors further highlighted a perceived lack of transparency surrounding the prescribing decisions by senior members of staff, which meant that the reason and rationale for antimicrobial prescribing was not always clear to them as they lacked context. This in turn contributed further to the uncertainty of the review process and inhibited deprescribing.

“Then you can run yourself into trouble when you try and work out why we’ve actually started these antibiotics, and it doesn’t bear any resemblance to what the guidelines say.”

Participant 5, junior doctor

Another issue contributing to uncertainty among newly qualified doctors (whose decisions tended to be guideline driven) was that antimicrobial guidelines were often perceived as being too vague, not comprehensive and not covering complex cases. As a result, they were unsure on options they were unfamiliar with.

“There are good resources that we have, so we have the antimicrobial guidelines. And they’re really good for treating empirically, but when it comes to…we know which organism, we’ve identified the organism, so then we’re trying to focus on antibiotics, they’re not as great at doing that. So that’s when we have to make our own decisions, and that’s when we end up usually just calling the microbiologists to see exactly what we need to do.”

Participant 2, clinical research fellow

“Our guidelines are so basic, they just say, do this if they’ve got this, but they don’t really help us to go, what happens when we don’t have results.”

Participant 3, clinical pharmacist

### Prescribing as a Set of Activities Enacted by Multiple Actors

We observed that prescribing and review was not one linear activity, but a complex set of actions of multiple actors participating in the patient journey. These included consultants and other doctors who prescribed, monitored, and reviewed antimicrobials, pharmacists reviewing prescriptions for accuracy and appropriateness, microbiologists analyzing cultures and advising, and nurses administering antimicrobials. All actors only had a partial view of the prescribing and review process. For example, those prescribing often did not make the initial decision to initiate antimicrobials (contributing to the uncertainty described above) [22].

Clinicians were struggling to fulfill various competing sets of priorities depending on their role. For example, busy doctors reported not always taking time to review antimicrobials and prescribing antimicrobials without looking at guidelines (both local and national) as this increased workloads, or not having time to ring the microbiology laboratory for results. Some also reported that stop dates were not set as they were unlikely to get noticed and actioned by busy staff [23].

“I think with like just about everything in the NHS, time is a big one. And I think it can be really, really easy to think, oh, I know what I prescribed for someone the last time they had a urinary tract infection… instead of looking at your guidelines every single time and making sure you’ve checked there’s not been a change since the last time.”

Participant 7, junior doctor

ePrescribing brought diverse actors together in a virtual environment, creating a shared decision space that often required actors to pick up actions initiated by others. However, every individual had different informational requirements and priorities at any point in time. This meant that processes initiated by 1 actor were not always followed up by others.

“In that text bit there’s also stop/start dates, indications, there are various other things, maybe special restrictions. It’s quite busy and I never check the review date that someone sets.”

Participant 2, clinical research fellow

### Hierarchies and Relationships Shaping Prescribing and Review Practices

The uncertainty around the initial prescribing decision was exacerbated by a reported reluctance of junior doctors to change or challenge a consultant’s prescribing decision at review.

“So there’s no professional practice whereby people, the nursing staff, me, the junior doctors are taught to say [consultant], do you know that this patient is on antibiotics and has been on them for ten days?”

Participant 11, consultant

“So when I worked on the admissions unit, there are a lot of patients who come from A&E who have been prescribed antibiotics and we don’t quite understand why. But as a junior doctor, maybe don’t feel empowered, if you know that the person who has seen them in A&E is the consultant or registrar and they’ve started antibiotics. As a more junior member of the medical team, we don’t feel empowered to say, do you actually think this person needs antibiotics?”

Participant 2, clinical research fellow

“Sometimes it’s not clear, I guess, you’d think some things are the right thing to do but it might not necessarily be that decision made by your senior consultant or team, you know, that they might not necessarily agree with that. So you would potentially continue with the antibiotics that are already prescribed or not step them down because you’re worried about someone else disagreeing with it.”

Participant 9, specialty training

Similarly, although the interaction between pharmacists and doctors was perceived to be very helpful for effective prescribing and medication review, it was not always straightforward. For example, pharmacists were in some instances reluctant to correct doctors, and tended to prefer finding ways of having a conversation with them that did not involve pointing out mistakes.

“I have to literally be like, did you mean to do that? Can you now change it? And then you have to nag them about half an hour later, can you make that change, can you make that change?”

Participant 3, clinical pharmacist

There was also an issue around responsibility, given the multitude of actors involved in the prescribing and review process. The current organization of clinical care and care pathways meant that no one single person or profession was responsible for review. Making a single person responsible would be difficult as staff rotated through complex shift patterns and might not be available to carry out review at the required time. This created a situation of temporary and rotating responsibility, where individual doctors had responsibility for the prescription they signed, but were not necessarily responsible for review and subsequent deprescribing decisions.

The issue of temporary responsibility was exacerbated because the technology logged who had accessed a case of the prescribing system, thereby creating a tacit responsibility to rectify any errors recorded. As a result, some pharmacists felt inhibited from accessing the system in certain situations where they were unable to take on this responsibility (eg, during the last hour of their shift when they did not want to take on extra work).

We also observed marked differences between wards in AMS practices. For instance, wards with a strong focus on infectious diseases benefitted greatly from strong existing relationships between doctors and microbiology staff, while in other specialties and more general wards with less use of antimicrobials these links were weaker.

“I think the inpatient support that we have is fantastic, so the close links that we have with the microbiology does mean that people are getting the right antibiotics, resistance has been avoided, people are starting and stopping at the right times.”

Participant 16, consultant

“But I think now for example especially looking after patients with infective endocarditis and cardiology because it is so much about making sure their infection is getting better, you are constantly thinking about their antibiotics and if they are on optimal antimicrobial therapy and you are also wanting input from the rest of the multidisciplinary team because we are constantly on the phone to microbiology about patients who have endocarditis.”

Participant 7, junior doctor

“So, we have a standard approach that all patients will get protocolised antibiotics, if their temperature doesn’t settle, then we’ve got a protocol about how we change those antibiotics and obviously we send blood cultures on everybody and if we get a positive blood culture, then with discussion with our microbiology team, we would then change those antibiotics.”

Participant 14, consultant

## Discussion

### Summary of Findings

We have described the antimicrobial prescribing and review process as a complex flow of activities undertaken by a range of actors differing in their levels of expertise or experience and specialism. The process was characterized by uncertainty around other clinicians’ intentions including the rationale for prescribing. The willingness to stop or change an existing prescription was influenced by existing hierarchies, ways of working, and differences in the degree of multidisciplinary collaboration. Multidisciplinary communication, collaboration, and coordination promoted good AMS practices by reducing uncertainty.

### Strengths and Limitations

This work has provided important insights into the existing antimicrobial prescribing practices in a hospital setting. These go beyond technological and behavioral aspects surrounding individual prescribing decisions, to include socio-organizational dynamics of wider multidisciplinary relationships and hierarchies. Although studying a single setting allowed us to gain in-depth insights into existing complexities, there is now a need to test these across a wider range of settings with varying existing AMS practices, systems, and infrastructures. The setting we studied was relatively advanced in terms of antimicrobial prescribing practices and stewardship. Studying sites that are less technologically mature might uncover additional issues. We were also limited by the fact that, due to the ongoing COVID-19 pandemic, we could not observe practices and dynamics. The COVID-19 pandemic may also have altered the AMS landscape—there has been more remote working, less direct education around AMS, and in particular the intricacies of the system (eg, training regarding “possible” and “probable” infection and interpretation of this on review). This would have helped to uncover additional intricacies arising in interactions that may not have been recalled by individuals. We further could have studied the wider organizational environment in more detail. For instance, some participants mentioned a tension between perceived value of initiatives by management (which often centered around cost savings), and the difficulty of attaching value to improved practices that may in some instances result in increased costs [24].

### Integration of Findings With Existing Literature

Competing priorities and uncertainty of prescribers and reviewers around prescribing decisions have previously been demonstrated in qualitative studies exploring the contextual factors surrounding antimicrobial prescribing processes [21,25-27]. Similarly, interprofessional relationships and hierarchies in prescribing practices have been shown to impact AMS. For example, some recent qualitative work shows that hierarchies tended to prevent clear communication in relation to antimicrobial prescribing, particularly between consultants and other doctors due to a fear of being reprimanded, and among seniors about losing autonomy and ownership [28]. A qualitative study of junior doctors’ experiences of antimicrobial prescribing described a perceived difficulty in finding support and existing hierarchies, characterized by different and sometimes conflicting opinions of senior colleagues and experiences of being reprimanded [29]. This is likely to be exacerbated by differences in attitudes to guidelines among senior and junior clinicians. For example, a recent systematic review found that senior doctors ignored local policies and guidelines unless they had been involved in the development of guidelines relating to their specialty [30]. This has led to the suggestion that efforts need to focus on providing a safe environment for all doctors to ask questions and challenge prescribing behavior [30].

Although these factors are known to be important, they have not been systematically applied to the development and use of electronic systems to improve AMS practices. For example, the importance of giving a rationale for an antimicrobial prescription from the initial prescriber has consistently been highlighted [3,29,31]. Our findings show that electronic systems can, in some instances, increase uncertainty, as systems bring together a group of stakeholders who may be physically disconnected. This is reinforced by work showing how electronic systems can in some instances compromise safety by obstructing the creation of an integrated patient narrative [32]. Here, a quick check of why a prescription was initiated is often not possible within a busy work environment, and it is also hampered by a potential lack of face-to-face contact and personal relationships. Questioning a senior’s decisions is likely to be more difficult under these circumstances.

The issue surrounding competing priorities is likely to be more difficult to address, but electronic systems provide scope to include nudges that help prescribers understand wider population-wide indicators such as local resistance patterns and thereby help navigate difficult decisions and trade-offs [33].

Despite these difficulties and the potential of electronic systems to exacerbate issues associated with a multitude of actors working together in a web-based space, our findings also support the need for a multidisciplinary approach [34]. Here, we have shown that multidisciplinary input can mitigate uncertainty in some instances (eg, through pharmacist and microbiologist input).

### Implications for Policy, Practice, and Research

Many existing intervention designs do not sufficiently account for existing work practices and may, therefore, fail to reach their potential. By doing this work, and feeding back our findings into intervention development, we seek to mitigate for this risk when developing our own ePrescribing-based AMS intervention going forward.

Our work shows that the existing practices and multidisciplinary team relationships and hierarchies have a significant impact on prescribing and medication review processes and thus on AMS. Attempts to develop tools and interventions to support AMS have not given sufficient attention to the existing context, work organization, and information flows. Simply involving individual clinicians in developing and implementing new interventions is not sufficient as prescribing and review practices are shaped by the interaction of diverse players—clinicians with differing fields and levels of expertise or experience and other specialists from pharmacy and microbiology. Effective interaction and communication between these various actors and in particular the senior members of multidisciplinary teams can help to reduce uncertainties that characterize the prescribing and review process, particularly for newly qualified doctors, who require the backup of other professionals whom they trust (eg, pharmacists).

We therefore recommend that efforts to improve AMS practices focus on reducing uncertainty and promoting interprofessional collaboration through education and technological design. This may, for instance, include implementing functionality that provides an indication of the source of infection and clear step-down criteria and guidance. It may also helpfully include links to guidelines in the prescribing rationale.

To address issues surrounding hierarchies, there is a need to train all clinicians on how to challenge prescribing decisions, and to cultivate a culture in which senior clinicians feel comfortable being challenged by others. To mitigate competing priorities, prescribers need to be educated not only on the role of AMS in population health, but also on the adverse impact of antimicrobials for individuals (eg, the potentially serious side effects, the gut microbe disturbance after using antimicrobials) [21].

### Conclusions

ePrescribing systems have significant potential to improve AMS, but their design needs to pay attention to the existing hierarchies, seek to reduce uncertainty, and improve communication between diverse clinician groups and health care professionals involved in initiating and reviewing prescriptions. Strengthening processes through organizational and technological components, including a focus on multidisciplinary teams and educational components, will need to play a crucial role in addressing these issues.

## Appendix

Multimedia Appendix 1 Coding Framework.

## Abbreviations

AMS: antimicrobial stewardship
CDS: clinical decision support
CPOE: computerized physician order entry
NHS: National Health Service

## Data Availability

Data are available on request.
