# Supplementary material for: ePrescribing-Based Antimicrobial Stewardship Practices in an English National Health Service Hospital: Qualitative Interview Study Among Medical Prescribers and Pharmacists
Source: JMIR Form Res. 2023 Jun 6;7:e37863. doi: 10.2196/37863 (PMC10282906; doi:10.2196/37863)
Supplement: Multimedia Appendix 1 [file formative_v7i1e37863_app1.docx]

Appendix 1 - Coding framework

| Antimicrobial resistance |
| --- |
| Antimicrobial stewardship |
| Antimicrobial stewardship management view |
| Antimicrobial guidelines |
| Audit |
| Barriers |
| Case-by-case |
| Education, learning |
| In current system |
| Multidisciplinary team |
| Antibiotic Review Kit (ARK) |
| Audit |
| Implementation |
| *Clostridioides difficile* and *Methicillin-resistant Staphylococcus aureus* (MRSA) |
| Clinical presentation |
| Consultant preferences |
| Rationale |
| COVID-19 |
| C-reactive protein test |
| Changes in antimicrobial use |
| COVID-19 antimicrobial guidelines |
| Long-term impact |
| Negative change |
| Positive change |
| Short-term impact |
| Education |
| Electronic Prescribing |
| Benefits |
| Decision making |
| Disbenefits |
| Default timings |
| Dialogue box |
| Lack of review alert |
| Personalised layouts |
| Review alert |
| Role-based access |
| Selection errors |
| Functions |
| Order sets |
| Paper to electronic Prescribing |
| Review date stop date |
| Prescribing room |
| Funding |
| Primary care records |
| Guidelines |
| Local guidelines |
| National guidelines |
| Hard stops |
| Health information exchange and interoperability |
| Implementation |
| Indication |
| Infectious diseases speciality |
| Initial prescribing |
| Interpersonal relationships |
| Information technology support |
| Leadership |
| Local configuration |
| Microbiology department |
| Advice |
| Delayed lab results |
| Unable to grow cultures |
| Variable lab processes |
| Microbiology tests & results |
| Nights and weekends |
| Nurses role |
| Overview dashboard |
| Patient safety |
| Pharmacist advice |
| Pop-up alerts |
| Problem points |
| Blood cultures |
| Clinical hierarchy |
| Clinical uncertainty |
| Clinician priorities |
| Culture, legacy practices |
| Forget to review |
| In-patient |
| Information technology infrastructure |
| Lack of knowledge |
| Lack of rationale |
| Management view |
| Patient discharge |
| Pharmacist review |
| Procalcitonin test to detect sepsis |
| Review |
| Consultants |
| Intravenous to oral, parenteral |
| Junior doctors |
| Lack of confidence |
| Learning |
| Pharmacist review |
| Site of infection query |
| Suggested improvements |
| Additional education |
| Antimicrobials ward overview |
| Clinician design |
| Compulsory review date |
| Continual policy update |
| Indication |
| In-system antimicrobial guidelines |
| In-system formulary |
| In-system decision aids |
| In-system duration guidelines |
| Public education |
| Review prompt |
| Time needed |
| Time saved |
| Ward rounds |
| Workarounds |
| Workflows |
